# Psychosocial risks: main threats to health care workers caused by the COVID-19 pandemic

**DOI:** 10.47626/1679-4435-2022-850

**Published:** 2022-03-30

**Authors:** Miguel Valencia-Contrera, Sandra Valenzuela-Suazo

**Affiliations:** 1 Programa de Magister en Enfermería, Universidad de Concepción, Concepción, Región del Biobío, Chile.; 2 Programa de Doctorado en Ciencia de Enfermería, Universidad Andrés Bello, Santiago, Región Metropolitana, Chile.; 3 Programa de Doctorado en Enfermería, Universidad de Concepción, Concepción, Región del Biobío, Chile.

**Keywords:** psychosocial impact, occupational health, coronavirus infections, occupational risks

## Abstract

Psychosocial risks circumscribe a relationship between the individual and the environment where they work, including from physical, social and organizational aspects of work, which, depending on personal capacities, can be potentially harmful to the health of workers. The entire world is currently witnessing one of the greatest health crises of the 21^st^ century, due to a new type of disease-causing virus called severe acute respiratory syndrome coronavirus 2 (SARS-CoV-2). The study aimed to survey the importance of psychosocial risks in health care professionals, based on evidence collected in the context of the COVID-19 pandemic. This is an analytical article whose guiding question was: what sources of psychosocial risks are present in health care workers during the COVID-19 pandemic? A total of 29 documents were included, coming from different sources of information that enriched the worked sample. The presence of the sources of psychosocial risks in the COVID-19 pandemic was analyzed, according to job content, workload and work pace, work schedule, control, environment and equipment, organizational structure and function, and role in the organization interpersonal relationships, career development, and work-life interface; furthermore, examples of situations that account for the presence of these risks are presented. All sources of psychosocial risks are present during the pandemic, some of their unfortunate harmful consequences have been currently described, and a call is thus made to address the problem.

## Introduction

In order to fully understand occupational risks, there is the need to define what psychological factors are, because it is imperative to become aware of them so as to get introduced to the essence of the concept. Such factors circumscribe a relationship between the individual and the environment where they work; currently, this environment is the work environment of health care professionals, including physical, social and organizational aspects that, depending on personal capacities, have an impact on workers’ health. When these factors may be potentially harmful, they are named psychosocial risks,^1^ although some currents of knowledge estimate that psychosocial risks are complex, and no definition has been accepted yet.^2^

In view of the complexity of psychosocial risks, one inevitably wonders about their etiology, which could be an essential factor for approaching these risks; furthermore, there is no doubt that these risks have been increasingly described by the scientific community over time. The World Health Organization (WHO) summarizes these risks in 10 categories:^3^ 1. Job content; 2. Workload and work pace; 3. Work schedule; 4. Control; 5. Environment and equipment; 6. Organizational culture and function; 7. Interpersonal relationships at work; 8. Role in organization; 9. Career development; 10. Home-work interface.

Currently, whole world is witnessing one of the greatest health crises of the 21^st^ century, due to a new type of pneumonia whose etiology is a new coronavirus named *severe acute respiratory syndrome coronavirus 2* (SARS-CoV-2), the causative pathogen of COVID-19,^4^ which reached such a worldwide spread that was characterized as a pandemic by the WHO general director on March 11, 2020.^5^

In light of the foregoing, the present article is developed aiming to survey the importance of psychosocial risks in the health team, based on evidence collected in the context of COVID-19 pandemic.

## Methodology

This is an analytical article whose guiding question was: what sources of psychosocial risks are present among health care workers during the COVID-19 pandemic? In order to answer this question, different sources of information enriched the worked sample, such as: guidelines from the WHO and Pan American Health Organization, government reports, scientific articles, newspaper articles, and books.

Finally, 29 documents were included, of which 12 were published in 2020,^7,14,17,20,22,23,27-29,32-34^ and 17 were published in 2021.^6,8-13,15,16,18,19,21,24-26,30,31^ The country of origin of information were the following: Spain,^6,8,24,32,33^ Brazil,^7,18^ the United States,^9^ Colombia,^10,14^ Costa Rica,^11^ Guatemala,^12^ United Kingdom,^13^ Chile,^15,16,23,26,27,29,34^ Argentina,^17^ Switzerland,^19,20,25,28^ Germany,^21^ Mexico,^22^ El Salvador,^30^ and Cuba.^31^

## Analysis

Next, we describe the different documented situations that account for the presence of the mentioned risks, which will be analyzed according to their possible etiology. Although certainly not all of these risks are present in all realities of health workers, at least one of them has been described as a consequence of COVID-19, hence the importance of approaching them.

### Job content

Due to the recent emergence of the crisis, the world population faces a problem of relatively unknown nature and, despite major efforts of the scientific community in understanding COVID-19, this disease continues to raise questions.^6^ Furthermore, it is worth considering that health team’s work involves continuous contact with people, many of whom are experiencing a process of suffering,^7^ a situation exacerbated in the current context and that certainly means a greater focus on psychosocial risks.

### Workload and work pace

An important aspect in the current scenario is the excessive workload and the higher pressure experienced by health care providers,^8^ who have to cope with one of the worst pandemics in history. A qualitative study recently conducted in Iran^9^ describes, in one of the subthemes emerging from semi-structured interviews with health care professionals, how most participants reported an overwhelming workload. Moreover, news with heartbreaking titles, such as: “Hospitals in Colombia are stretched to the limit due to COVID-19,”^10^ “Costa Rica continues to report crowded hospitals and high rates of COVID infection,”^11^ “With crowded hospitals, Guatemala faces a new health crisis due to COVID-19, warns the ministry of Health,”^12^ or “COVID-19 in the United Kingdom: It has been one of the worst shift in my life,”^13^ are some of the current examples presented worldwide that illustrate workload and work pace as a psychosocial risk.

### Work schedule

As an essential service for society, health care the creation of strategies to maintain its provision 24 hours a day, 365 days a year, which is translated into the need for workers to perform night shifts and has gained special importance in the current context, because workers often have had to adapt to extended shifts, consisting of 24-hour continuous shifts. Alcover^14^ describes the high pressures to which workers have been exposed as one of the consequences of the pandemic, pressures that contribute to productivity and are translated into increased working hours and undertaking of tasks, functions or roles that exceed the usual ones in their position. Additionally, titles in mass medias, such as: “We are not machines, we are people,”^15^ “36-hour shifts, students, retired physicians, and those from other specialties: strategies that health care centers have been using to populate ICUs,”^16^ underline the vulnerability in relation to work schedule of the health team.

### Control

As previously mentioned, the workload of health care workers has been extremely high, although the most complicated aspect of the scenario is the impossibility of controlling this load, in an unprecedented health crisis, which forced people to live in uncentainty,^17^ with a constant fear of getting infected,^18^ Although over time the scientific community has made tremendous efforts, with great results in a short period of time,^19^ there are still questions to addressed, increased rates of cases, or others services to offer to society. It is worth remembering that COVID-19 is not the only battle to be fought. Instead, it is a problem that should be added to the list of health problems to coped with, since recently the WHO has informed that heart diseases are still the leading cause of mortality worldwide.^20^

### Environment and equipment

In a hostile context such as the current one, the worst-case scenario is a lack of appropriate resources to fight against the pandemic. However, this unfortunately is the reality of many countries,^21^ as well lack of space in points of care,^22,23^ portraying the poor environmental conditions experienced by health care professionals, who also had to deal with field hospitals,^24^ which consist of adaptations intended to respond to patient overcrowding but not to the need for a workplace that complies with workers’ needs.

### Organizational culture and function, and role in the organization

A key aspect to mitigate hostility in the current context is presenting good communication and support in solving problems, an aspect that takes on special importance in the tactical and strategic levels of health institutions. However, it has been neglected in different realities, with studies showing nurses’ complaints on work, organizational and support relationships, organizational preparedness, and occupational safety.^25^ Furthermore, news such as “Suicidal of a young nurse: claims say she had suffered workplace harassment in Hospital Clínico Viña del Mar,”^26^ or “Director is accused: health care workers in Osorno denounce malpractices in Hospital de Río Negro”^27^ reveal a great neglected need.

### Interpersonal relationships

According to the International Labor Organization (ILO),^28^ work relationships continue to be the cornerstone of labor protection. However, this element is currently complex to potentiate, because the recommended social distancing could impose a barrier, in addition to other factors, such as the abovementioned overload or high pressure in work activities. Conversely, interpersonal relationships could be improved in those who had to adopt teleworking.

### Career development

Due to the great demand for health care professionals to face the health crisis, there was an increase in the number of hired workers. Nonetheless, situations of job insecurity were described, and news such as the following often appeared in the mass media: “I am afraid of getting fired tomorrow: Reports of those who fight against COVID-19 in poor work conditions,”^29^ or “Job instability concerns health care professionals hired due to COVID-19 emergency.”^30^ This insecurity resulted from the fact that many professionals recently hired by health institutions, because of their type of employment contract, were exempt from essential benefits in a context of high occupational risks, such as: no right to annual vacations, social insurance, health pension, or medical leave.

### Home-work interface

An aspect that has changed everybody’s life was the fear of COVID-19 infection.^31^ Because of their high level of exposure, many health care professionals preferred to remain distant from their loved ones, especially from those belonging to the high-risk age groups. However, the saddest aspect of this situation is how fear may turn into discrimination and rejection by the community towards the health team. Many titles of news in the mass media described situations that put it into evidence: “As long as it lasts, I ask you to considering moving out,”^32^ “The indignation of a nurse when suffering rejection from her neighbors: It is hypocritical of them not wanting in the building but then applauding health care professionals,”^33^ or “Puerto Montt: Medical College opposes to discrimination against health care workers in the midst of the COVID-19 pandamic.”^34^

The unfortunate situations that account for exposure of health care workers to psychosocial risks led to results as unfortunate as the death of a person’s life. The consequences of these situations are described next.

### Consequences of psychosocial risks

**Figure 1:**
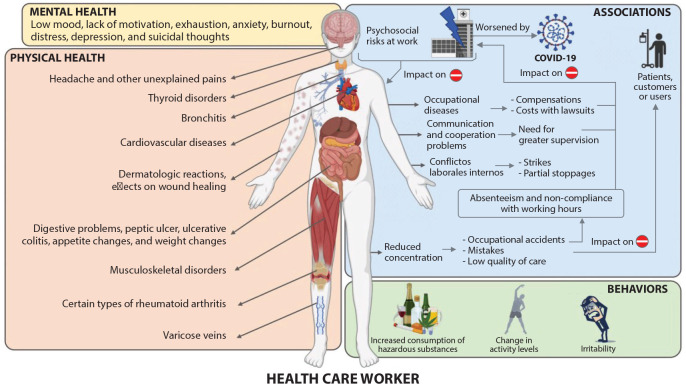
Consequences of psychosocial risks.

The ILO,^35^ the WHO,^3,36^ and authors such as Neffa^2,37^ have contributed in understanding psychosocial risks, showing a myriad of both direct and indirect consequences to workers’ health, encompassing mental health and physical integrity problems, occupational diseases, social problems, behavioral changes, and impacts on the workplace; with didactic purposes to facilitate understanding, Figure 1 summarizes the broad range of consequences and associations of psychological risks.

It is no news that maintaining stressful factors for a long time leads to severe health problems when time to recover and regenerate is short.^38^ Evidence shows that the COVID-19 pandemic leads a significant psychosocial load to health care professionals, especially nurses and women, affecting both frontline and non-frontline workers.^39^

Next, without being exhaustive, we present some studies that already demonstrate the presence of the consequences of psychological risks derived from the COVID-19 pandemic. A study developed in India^40^ concluded that most anesthesiologists on COVID-19 duty suffer from some degree of anxiety and insomnia. Another study in China^41^ showed that frontline health care workers had a significant psychosocial distress. In the United States, a study^42^ found that nurses and other health care providers consistently reported increased anxiety during the pandemic. In Iran, a study^43^ showed that the COVID-19 pandemic has significant consequences for the mental health of nurses. Another study in China^44^ observed high anxiety levels among the dental staff. A study in Latin America^45^ concluded that spread of COVID-19 pandemic has negatively impacted the professional, financial, and psychosocial health of orthopedic trauma surgeons. Finally, a study in Singapur^46^ noted a high prevalence of depression, anxiety and stress among frontline pediatric health care workers during the COVID-19 pandemic.

## Final considerations

The present study provided evidence on the presence of sources of psychological risks, in their different forms, in the current context of COVID-19 pandemic, increasing the vulnerabilities of the health teams that had to face one of the greatest health crises of the 21^st^ century. There were reports of multiple effects derived from exposure to psychosocial risks in non-pandemic conditions, encompassing from physical and mental health to workers’ social dimension. Therefore, the early control of these risks is especially important in the current context, and a call is made to address the problem, because its unfortunate consequences have already been reported. According to the authors of the present study, such approach is the greatest current challenge of occupational health.
